# Body composition change indices combined with Prognostic Nutritional Index predicts the clinical outcomes of patients with gastric cancer treated with immune checkpoint inhibitor

**DOI:** 10.1002/cam4.7110

**Published:** 2024-03-20

**Authors:** Guiming Deng, Dayong Zhu, Zhongze Du, Yingwei Xue, Hongjiang Song, Yuanzhou Li

**Affiliations:** ^1^ Department of Gastrointestinal Surgery Harbin Medical University Cancer Hospital, Harbin Medical University Harbin Heilongjiang China; ^2^ Department of General Surgery Heilongjiang Provincial Hospital Harbin Heilongjiang China; ^3^ Department of Radiology Harbin Medical University, Cancer Hospital, Harbin Medical University Harbin Heilongjiang China

**Keywords:** fat area, gastric cancer, PD‐1/PD‐L1, prognosis, Prognostic Nutritional Index, skeletal muscle mass index

## Abstract

**Objective:**

This study aimed to investigate the prognostic significance of the Prognostic Nutritional Index (PNI) in conjunction with body composition change indices, namely subcutaneous fat area (SFA) and skeletal muscle index (SMI), with regard to clinical outcomes in patients with gastric cancer (GC) undergoing immune checkpoint inhibitors (ICIs) treatment.

**Methods:**

This retrospective investigation encompassed patients with comprehensive clinical and pathological data, inclusive of portal phase enhanced CT images. Continuous variables underwent analysis utilizing the Student *t*‐test or Mann–Whitney *U*‐test, while categorical variables were assessed employing the Pearson chi‐squared test or Fisher test. Survival outcomes were evaluated using Kaplan–Meier survival curves and the Log‐rank test. Independent prognostic indicators were determined through Cox regression analysis, and a nomogram predicting survival probability for progression‐free survival (PFS) and overall survival (OS) was constructed.

**Results:**

Within the PNI‐SFA groups, patients in Group 1 exhibited inferior PFS and OS compared to the other two groups. Similarly, among the PNI‐SMI groups, Group 1 patients demonstrated poorer PFS and OS. PNI‐SMI and Eosi were identified as independent prognostic factors through Cox regression analysis. Furthermore, positive associations with patient prognosis were observed for BMI, SAF, SMI, and PNI.

**Conclusion:**

The comprehensive consideration of PNI‐SFA and PNI‐SMI proved to be a superior prognostic predictor for GC patients undergoing ICI treatment.

## 1 INTRODUCTION

Gastric cancer (GC), ranking as the fifth most frequently diagnosed malignancy globally and the third leading cause of cancer‐related mortality,^1^ exhibits a notable gender disparity, being twice as prevalent in men compared to women.^2^ The incidence of GC demonstrates an association with countries characterized by a high Human Development Index (HDI), with *Helicobacter pylori* infection prevalence further influencing its occurrence. East Asia, marked by elevated HDI and the prevalence of virulent *H*. *pylori* strains, bears a substantial burden of GC.^3^ Immunosuppressant therapy has emerged as the primary treatment for GC, particularly benefiting patients with advanced stages.^4^ Prognostic biomarkers such as PD‐1/PD‐L1 expression levels and microsatellite instability (MSI) have been identified for individuals undergoing ICIs treatment.^5^, ^6^, ^7^ Alongside disease status, nutritional considerations significantly impact the prognosis and treatment outcomes of GC patients. This study centers on evaluating the predictive capacity of body composition and blood indicators reflecting nutritional status in GC patients treated with ICIs.

Factors influencing patient nutritional status include decreased appetite and malaise.^8^, ^9^ Cancer‐associated cachexia, characterized by weight loss and specific skeletal muscle and adipose tissue depletion, is prevalent in GC patients.^10^ Malnutrition not only accelerates cancer progression but also hampers treatment efficacy.^11^, ^12^ While body mass index (BMI) serves as an objective nutritional assessment, aiding in diagnosing malignant stasis alongside weight loss rates, it does not discern the distribution of visceral and subcutaneous fat.^13^, ^14^ BMI is commonly employed to predict cancer risk;^15^ however, its prognostic utility for cancer outcomes exhibits disparities, as illustrated in a systematic evaluation and meta‐analysis demonstrating an association between high BMI and increased colorectal cancer‐specific and overall mortality.^16^ This suggests obesity's significant correlation with heightened recurrence and progression risk, serving as a crucial indicator of cancer prognosis. The PNI, reflecting nutritional inflammation in the body, has been established as an indicator of the nutritional status of GC patients in multiple studies.^17^, ^18^

Several studies have utilized CT images to calculate the fat and muscle areas of the third lumbar vertebra, examining the impact of abdominal fat and muscle loss on cancer prognosis. For instance, in GC, muscle loss is linked to a poorer prognosis,^19^ while differences in abdominal fat distribution correlate with the response to intravesical Bacillus Calmette‐Guerin immunotherapy.^20^

This study aims to analyze the prognostic value of PNI, BMI, and the third lumbar vertebral fat and muscle areas in patients with GC treated with ICIs, encompassing a hematological to body composition synthesis perspective.

## 2 MATERIALS AND METHODS

### 2.1 Patients

A total of 124 GC (GC) patients who underwent immunotherapy with immune checkpoint inhibitors (ICIs) at our institution between February 2016 and October 2022 were included in this study. Patient data, encompassing clinical and tumor characteristics, laboratory parameters, and portal phase CT images, were retrieved from medical records. Approval from the Institutional Review Board was obtained for this retrospective study, and due to its retrospective nature, the requirement for informed consent was waived. The ICIs immunotherapies comprised antibodies targeting programmed cell death protein 1 (PD‐1), namely anti‐PD‐1 antibodies, anti‐programmed death ligand 1 (PD‐L1) antibodies, and combination regimens involving ICIs. Exclusion criteria comprised (1) absence of pre‐treatment CT scans, (2) premature discontinuation of treatment, and (3) incomplete clinical data.

### 2.2 Data collection

The study endpoints were PFS and OS, determined through telephone follow‐up, with the last follow‐up conducted in December 2022. OS and PFS were calculated from the initiation of treatment to the last follow‐up, with disease progression identified primarily through enhanced CT scans. In the absence of evidence of disease progression, the last follow‐up time was considered as PFS.

### 2.3 Fat area, skeletal muscle area, and PNI

A radiologist with 15 years of imaging experience, blinded to clinical outcomes, analyzed CT images of all patients. The Prognostic Nutritional Index (PNI) was calculated as albumin level (g/L) + 5 × lymphocyte count (10^9/L). Cross‐sectional images at the third lumbar vertebra level were extracted from pre‐treatment CT images. Visceral fat area (VFA), subcutaneous fat area (SFA), and skeletal muscle area (SMA) were measured using a 3D slicer. Adipose tissue and skeletal muscle tissue were differentiated based on preset hounsfield unit (HU) thresholds. Total fat area (TFA) was computed as the sum of VFA and SFA, and the skeletal muscle index (SMI) was determined as SMA/height^^2^. Receiver Operating Characteristic (ROC) analysis established optimal cutoff values for dichotomizing TFA, SAF, VFA, SMI, BMI, and PNI. The best cutoff values for TFA, SAF, VFA, and SMI were 192.98, 96.87, 173.25, and 27.36 for male students. For female students, the best cutoff values for TFA, SAF, VFA, and SMI were 213.77, 127.36, 50.25, and 31.10, respectively. The best cutoff value for PNI was 48.13 and for BMI the best cutoff value was 20.80. Group categorization for PNI‐SFA was determined by PNI and SFA values, with Group 1 having PNI <48.13 and SFA below the optimal cutoff, Group 3 having PNI ≥48.13 and SFA greater than or equal to the optimal cutoff, and all other cases falling into Group 2. Gastrointestinal surgeons trained in 3D slicer verification ensured data accuracy by averaging the results.

### 2.4 Statistical analysis

Normally distributed data were presented as mean ± standard deviation (SD), and non‐normally distributed data as median (minimum and maximum values). Continuous variables underwent analysis using Student *t*‐test or Mann–Whitney *U*‐test, while categorical variables were assessed using Pearson chi‐squared test or Fisher test. Kaplan–Meier survival curves and log‐rank test evaluated survival outcomes between different group, and multivariate Cox's regression analysis incorporated meaningful metrics from univariate analysis to identify independent prognostic factors associated with OS and PFS. These factors were used to establish predictive models for OS and PFS. R 4.1.3 (Vienna, Austria) and SPSS 25.0 (Chicago, IL, USA) were employed for data analysis, with a two‐sided *p*‐value <0.05 considered statistically significant.

## 3 RESULTS

### 3.1 Patient characteristics

The study enrolled a total of 124 GC patients undergoing ICIs treatment, comprising 96 (77.4%) males and 28 (22.6%) females. Among the patient cohort, 50 individuals (40.3%) were administered PD‐1 concurrently with SOX, while 38 (30.7%) received PD‐1 alongside XELOX, and 36 (29.0%) were subjected to PD‐1 in combination with other chemotherapeutic protocols. Specifically, 69 patients (55.6%) underwent first‐line PD‐1 treatment, and 55 patients (44.4%) received PD‐1 as a second‐line intervention. Notably, within this cohort, 49 patients (49.5%) underwent surgical procedures during the course of their treatment. Because of the non‐normal distribution observed in tumor marker levels, patients were stratified into two groups using the median values of the respective tumor markers. Significant statistical differences were observed in age (*p* = 0.041), BMI (*p* < 0.001), carbohydrate antigen 724 (CA724) (*p* < 0.001), SFA (*p* < 0.001), VFA (*p* < 0.001), and TFA (*p* < 0.001) among distinct PNI‐SFA groups. Conversely, age (*p* = 0.046) and SMI (*p* = 0.015) demonstrated statistical differences among distinct PNI‐SMI groups (Table 1).

**TABLE 1:** Patient characteristics.

Item, mean (SD)	PNI‐SAF			*p* value	PNI‐SMI			*p* value
	Group 1	Group 2	Group 3		Group 1	Group 2	Group 3	
	*n* = 58	*n* = 38	*n* = 28		*n* = 21	*n* = 66	*n* = 37	
Age	59.31 (8.60)	54.16 (10.67)	57.71 (7.53)	0.041	60.29 (9.79)	58.17 (7.89)	54.30 (10.59)	0.046
BMI	21.73 (3.04)	20.48 (4.01)	24.98 (2.59)	<0.001	22.89 (3.90)	21.39 (3.85)	22.84 (3.90)	0.083
Sex (%)				0.291				0.291
Male	45 (77.6)	32 (84.2)	19 (67.9)		16 (76.2)	48 (72.7)	32 (86.5)	
Female	13 (22.4)	6 (15.8)	9 (32.1)		5 (23.8)	18 (27.3)	5 (13.5)	
Surgery				0.091				0.297
Yes	17 (29.3)	19 (50.0)	13 (46.4)		9 (42.9)	22 (33.3)	18 (48.6)	
No	41 (70.7)	19 (50.0)	15 (53.6)		12 (57.1)	44 (66.7)	19 (51.4)	
Primary tumor site (%)				0.966				0.163
Upper 1/3	9 (15.6)	7 (18.5)	5 (17.9)		6 (28.6)	8 (12.1)	7 (18.9)	
Middle 1/3	14 (24.1)	11 (28.9)	5 (17.9)		3 (14.3)	14 (21.2)	13 (35.1)	
Low 1/3	33 (56.9)	19 (50.0)	17 (60.6)		12 (57.1)	40 (60.6)	17 (45.0)	
Whole	2 (3.4)	1 (2.6)	1 (3.6)		0 (0.0)	4 (6.1)	0 (0.0)	
Pathology (%)				0.135				0.291
Adenocarcinoma	14 (24.1)	11 (28.9)	10 (35.7)		8 (38.1)	18 (27.3)	9 (24.3)	
Others	3 (5.2)	7 (18.5)	1 (3.6)		0 (0.0)	5 (7.6)	6 (16.2)	
Unknown	41 (70.7)	20 (52.6)	17 (60.7)		13 (61.9)	43 (65.2)	22 (59.5)	
TNM stage (%)				0.687				0.372
I	1 (1.7)	1 (2.6)	2 (7.1)		1 (4.8)	3 (4.5)	0 (0.0)	
II	2 (3.4)	2 (5.3)	1 (3.6)		2 (9.5)	1 (1.5)	2 (5.4)	
III	11 (19.0)	11 (28.9)	7 (25.0)		4 (19.0)	14 (21.2)	11 (29.7)	
IV	44 (75.9)	24 (63.2)	18 (64.3)		14 (66.7)	48 (72.7)	24 (64.9)	
PD‐1 (%)				0.450				0.222
Positive	3 (5.2)	5 (13.2)	2 (7.1)		1 (4.7)	6 (9.1)	3 (8.1)	
Negative	4 (6.9)	2 (5.2)	4 (14.3)		3 (14.3)	2 (3.0)	5 (13.5)	
Unknown	51 (87.9)	31 (81.6)	22 (78.6)		17 (81.0)	58 (87.9)	29 (78.4)	
PD‐L1 (%)				0.061				0.260
Positive	5 (8.6)	1 (2.6)	5 (17.9)		4 (19.0)	4 (6.1)	3 (8.1)	
Negative	2 (3.5)	6 (15.8)	2 (7.1)		1 (4.8)	4 (6.1)	5 (13.5)	
Unknown	51 (87.9)	31 (81.6)	21 (75.0)		16 (76.2)	58 (87.8)	29 (78.4)	
AFP (%)				0.900				0.474
<2.92 ng/mL	28 (48.3)	19 (50.0)	15 (53.6)		8 (38.1)	34 (51.5)	20 (54.1)	
≥2.92 ng/mL	30 (51.7)	19 (50.0)	13 (46.4)		13 (61.9)	32 (48.5)	17 (45.9)	
CEA (%)				0.070				0.963
<4.24 ng/mL	28 (48.3)	15 (39.5)	19 (67.9)		11 (52.4)	33 (50.0)	18 (48.6)	
≥4.24 ng/mL	30 (51.7)	23 (60.5)	9 (32.1)		10 (47.6)	33 (50.0)	19 (51.4)	
CA199 (%)				0.091				0.773
<17.63 U/L	27 (46.6)	16 (42.1)	19 (67.9)		12 (57.1)	32 (48.5)	18 (48.6)	
≥17.63 U/L	31 (53.4)	22 (57.9)	9 (32.1)		9 (42.9)	34 (51.5)	19 (51.4)	
CA724 (%)				<0.001				0.272
<4.40 U/L	19 (32.8)	20 (52.6)	22 (78.6)		7 (33.3)	34 (51.5)	20 (54.1)	
≥4.40 U/L	39 (67.2)	18 (47.4)	6 (21.4)		14 (66.7)	32 (48.5)	17 (45.9)	
CA125II (%)				0.386				0.276
<21.94 U/L	26 (44.8)	19 (50.0)	17 (60.7)		8 (38.1)	32 (48.5)	22 (59.5)	
≥21.94 U/L	32 (55.2)	19 (50.0)	11 (39.3)		13 (61.9)	34 (51.5)	15 (40.5)	
SMI (cm^2^/m^2^)	34.43 (8.63)	33.65 (10.69)	38.39 (9.01)	0.171	36.14 (10.25)	32.73 (8.88)	38.61 (9.10)	0.015
SAF (cm^2^)	62.46 (28.42)	59.27 (24.86)	138.66 (32.23)	<0.001	86.30 (50.35)	75.17 (42.23)	80.65 (40.29)	0.523
VAF (cm^2^)	64.48 (61.64)	57.71 (44.56)	117.07 (56.19)	<0.001	82.52 (72.32)	66.26 (47.89)	83.90 (56.58)	0.356
TAF (cm^2^)	126.94 (66.98)	116.98 (64.91)	255.73 (67.22)	<0.001	168.81 (100.92)	141.43 (78.45)	164.55 (90.53)	0.371

Abbreviations: BMI, body mass index; CA125II, carbohydrate antigen 125II; CA199, carbohydrate antigen 199; CA724, carbohydrate antigen 724; CEA, carcinoembryonic antigen; Others of Pathology, include mucinous carcinoma, signet ring cell carcinoma, mixed carcinoma; SFA, subcutaneous fat area; SMI, skeletal muscle index; TFA, Total fat; VFA, Visceral fat area.

### 3.2 Blood parameters

Analysis of blood parameters for all patients revealed significant correlations between total bilirubin (TBIL) (*p* = 0.001), direct bilirubin (DBIL) (*p* = 0.048), indirect bilirubin (IDBIL) (*p* = 0.001), total protein (TP) (*p* < 0.001), albumin (ALB) (*p* < 0.001), pre‐ALB (PALB) (*p* < 0.001), uric acid (UA) (*p* = 0.029), lymphocytes (Lym) (*p* < 0.001), hemoglobin (Hb) (*p* < 0.001), red blood cell count (RBC) (0.023), and platelet count (Plt) (0.019) with PNI‐SFA. Furthermore, TBIL (*p* = 0.014), IDBIL (*p* = 0.005), TP (*p* < 0.001), ALB (*p* < 0.001), PALB (*p* < 0.001), UA (*p* = 0.038), Lym (*p* = 0.001), Hb (*p* < 0.001), and RBC (*p* = 0.006) exhibited significant correlations with PNI‐SMI (Table 2).

**TABLE 2:** Patients' blood parameters.

Item, mean (SD)	PNI‐SAF			*p* value	PNI‐SMI			*p* value
	Group 1	Group 2	Group3		Group 1	Group 2	Group 3	
	*n* = 58	*n* = 38	*n* = 28		*n* = 21	*n* = 66	*n* = 37	
ALT (U/L)	20.18 (15.96)	24.21 (25.91)	21.33 (16.94)	0.657	19.91 (13.00)	20.38 (17.13)	25.00 (26.05)	0.240
AST (U/L)	27.45 (22.95)	25.18 (14.36)	24.07 (15.48)	0.711	23.33 (11.66)	27.24 (22.73)	25.27 (14.74)	0.618
γ‐GGT (U/L)	71.45 (138.15)	46.47 (76.97)	57.39 (70.10)	0.769	54.14 (84.57)	69.65 (128.25)	48.19 (79.98)	0.903
ALP (U/L)	121.09 (98.12)	109.16 (63.89)	100.52 (41.79)	0.945	103.52 (39.94)	117.69 (93.92)	109.30 (63.79)	0.895
TBIL (μmol/L)	12.83 (8.74)	15.66 (6.65)	16.74 (9.19)	0.001	11.97 (4.64)	13.97 (8.75)	17.15 (8.83)	0.014
DBIL (μmol/L)	3.28 (4.00)	3.05 (1.57)	3.72 (2.33)	0.048	2.49 (1.27)	3.58 (3.87)	3.30 (1.90)	0.219
IDBIL (μmol/L)	9.54 (5.16)	12.61 (5.46)	13.02 (7.56)	0.001	9.48 (3.48)	10.39 (5.53)	13.85 (7.26)	0.005
TP (g/L)	65.29 (6.69)	74.37 (5.58)	69.49 (6.90)	<0.001	65.43 (5.86)	67.35 (7.67)	74.01 (5.27)	<0.001
ALB (g/L)	35.54 (3.68)	43.65 (6.38)	39.74 (4.04)	<0.001	36.83 (3.03)	36.86 (4.47)	43.95 (6.36)	<0.001
GLOB (g/L)	29.75 (4.93)	31.73 (4.95)	29.66 (4.58)	0.103	28.63 (4.35)	30.46 (5.04)	31.09 (4.87)	0.186
PALB (g/L)	184.19 (60.38)	237.53 (66.47)	223.95 (67.84)	<0.001	204.33 (66.17)	189.18 (60.14)	248.19 (67.08)	<0.001
Urea (mmol/L)	5.93 (1.79)	6.35 (1.78)	5.57 (1.65)	0.091	6.05 (1.75)	5.83 (1.75)	6.19 (1.83)	0.539
CREA (μmol/L)	77.00 (18.48)	79.97 (16.24)	76.39 (13.94)	0.498	76.67 (16.71)	76.67 (17.24)	80.38 (16.24)	0.401
UA (μmol/L)	296.66 (85.81)	332.69 (94.29)	335.39 (98.84)	0.029	297.00 (63.06)	306.41 (92.01)	345.38 (102.72)	0.038
LDH (U/L)	262.59 (277.07)	200.00 (89.22)	197.79 (97.91)	0.700	237.86 (222.27)	243.32 (238.74)	197.68 (92.71)	0.971
WBC (10^9^/L)	7.73 (6.51)	7.37 (2.68)	6.68 (2.35)	0.713	7.60 (4.71)	7.43 (5.80)	7.18 (2.43)	0.924
NEU (10^9^/L)	4.96 (2.88)	4.81 (2.43)	4.00 (2.10)	0.196	5.53 (4.41)	4.53 (1.95)	4.51 (2.20)	0.720
Lym (10^9^/L)	1.31 (0.44)	1.86 (0.53)	1.95 (0.67)	<0.001	1.39 (0.46)	1.54 (0.61)	1.90 (0.56)	0.001
Mono (10^9^/L)	0.49 (0.19)	0.55 (0.26)	0.47 (0.16)	0.497	0.49 (0.21)	0.49 (0.19)	0.55 (0.25)	0.707
Eosi (10^9^/L)	0.13 (0.14)	0.11 (0.09)	0.13 (0.08)	0.442	0.11 (0.08)	0.12 (0.13)	0.13 (0.10)	0.522
Baso (10^9^/L)	0.03 (0.02)	0.02 (0.02)	0.02 (0.01)	0.721	0.02 (0.02)	0.03 (0.02)	0.02 (0.01)	0.756
Hb (g/L)	116.08 (25.77)	135.93 (21.93)	133.14 (20.72)	<0.001	117.10 (26.45)	120.49 (23.47)	140.95 (21.33)	<0.001
RBC (10^12^/L)	4.18 (0.73)	4.57 (0.65)	4.54 (0.64)	0.023	4.18 (0.71)	4.27 (0.70)	4.71 (0.61)	0.006
Plt (10^9^/L)	275.19 (97.12)	240.08 (90.90)	222.50 (74.14)	0.019	235.10 (84.44)	267.68 (94.81)	235.41 (90.27)	0.094
Fbg (g/L)	3.90 (1.08)	4.16 (3.89)	3.51 (1.27)	0.093	3.78 (1.03)	3.84 (1.18)	4.05 (3.96)	0.142
DDi (mg/L)	1.51(2.67)	1.10(2.01)	1.08(1.75)	0.275	1.14 (1.98)	1.60 (2.76)	0.81 (1.23)	0.074

Abbreviations: ALB, albumin; ALP, alkaline phosphatase; ALT, alanine transaminase; AST, aspartate aminotransferase; Baso, basocytes; CREA, creatinine; DBIL, direct bilirubin; DDi, D‐Dimer; Eosi, eosinophil; Fbg, fibrinogen; GLOB, globulin; Hb, hemoglobin; IDBIL, indirect bilirubin; LDH, lactate dehydrogenase; Lym, lymphocyte; Mono, monocytes; NEU, neutrophil; PALB, prealbumin; Plt, platelet; RBC, red blood cell; TBIL, total bilirubin; TP, total protein; UA, uric acid; Urea, urea nitrogen; WBC, white blood cell; γ‐GGT, γ‐glutamyl transferase.

### 3.3 Univariate and multivariate Cox's regression analysis

Univariate and multivariate Cox's proportional risk models were employed to analyze independent prognostic factors. All fat and muscle indicators were included in the univariate analysis, revealing that TP, PALB, Eosinophils (Eosi), CA724, carbohydrate antigen 125II (CA125II), BMI, PNI‐SFA, and PNI‐SMI (all *p* < 0.005) were prognostic factors for OS. In addition, alkaline phosphatase (ALP), TBIL, PALB, Eosi, CA724, CA125, BMI, PNI‐SFA, and PNI‐SMI (all *p* < 0.005) were prognostic factors for PFS. Subsequent multivariate Cox analysis identified Eosi and PNI‐SMI (all *p* < 0.005) as independent prognostic factors for both PFS and OS (Table 3).

**TABLE 3:** Univariate and multivariate analysis for PFS and OS.

	Univariate analysis	OS	Multivariate analysis		Univariate analysis	PFS	Multivariate analysis	
Parameters	Hazard ratio (95%CI)	*p* value	Hazard ratio (95%CI)	*p* value	Hazard ratio (95%CI)	*p* value	Hazard ratio (95%CI)	*p* value
ALT (U/L)	0.836 (0.489–1.428)	0.551			1.337 (0.782–2.286)	0.288		
AST (U/L)	1.883 (0.458–7.744)	0.380			2.478 (0.603–10.180)	0.208		
γ‐GGT (U/L)	0.978 (0.572–1.670)	0.934			0.994 (0.574–1.721)	0.982		
ALP (U/L)	1.676 (0.955–2.940)	0.072			1.983 (1.132–3.474)	0.017	1.535 (0.829–2.843)	0.173
TBIL (μmol/L)	1.555 (0.867–2.788)	0.138			2.143 (1.201–3.826)	0.010	1.769 (0.944–3.315)	0.075
DBIL (μmol/L)	1.326 (0.774–2.273)	0.305			1.459 (0.857–2.485)	0.164		
IDBIL (μmol/L)	1.382 (0.802–2.384)	0.244			1.927 (1.122–3.311)	0.017		
TP (g/L)	0.374 (0.159–0.876)	0.024	0.378 (0.140–1.020)	0.055	0.490 (0.210–1.145)	0.099		
ALB (g/L)	1.044 (0.582–1.874)	0.885			1.282 (0.715–2.298)	0.405		
GLOB (g/L)	0.497 (0.179–1.378)	0.179			0.456 (0.164–1.269)	0.133		
PALB (g/L)	0.529 (0.308–0.911)	0.022	0.685 (0.376–1.248)	0.216	0.556 (0.326–0.950)	0.032	0.705 (0.392–1.269)	0.244
Urea (mmol/L)	0.729 (0.384–1.385)	0.335			0.748 (0.392–1.429)	0.379		
CREA (μmol/L)	0.895 (0.509–1.571)	0.669			1.062 (0.608–1.856)	0.833		
UA (μmol/L)	0.740 (0.370–1.477)	0.393			0.780 (0.386–1.575)	0.488		
LDH (U/L)	1.037 (0.562–1.911)	0.908			1.000 (0.547–1.829)	0.999		
WBC (10^9^/L)	1.649 (0.944–2.881)	0.079			1.550 (0.890–2.698)	0.121		
NEU (10^9^/L)	1.516 (0.845–2.718)	0.163			1.454 (0.813–2.601)	0.207		
Lym (10^9^/L)	0.582 (0.331–1.042)	0.060			0.585 (0.332–1.031)	0.064		
Mono (10^9^/L)	0.789 (0.446–1.336)	0.378			0.807 (0.476–1.368)	0.426		
Eosi (10^9^/L)	3.478 (1.707–7.089)	0.001	2.915 (1.376–6.177)	0.005	2.973 (1.481–5.966)	0.002	3.050 (1.439–6.468)	0.004
Baso (10^9^/L)	1.146 (0.670–1.960)	0.618			1.436 (0.834–2.473)	0.192		
Hb (g/L)	1.068 (0.624–1.827)	0.810			1.050 (0.615–1.793)	0.858		
RBC (10^12^/L)	1.050 (0.618–1.786)	0.856			1.051 (0.617–1.789)	0.856		
Plt (10^9^/L)	0.646 (0.357–1.166)	0.147			0.634 (0.350–1.148)	0.133		
Fbg (g/L)	0.045 (0.000–12.645)	0.280			0.044 (0.000–7.540)	0.234		
DDi (mg/L)	1.875 (0.970–3.625)	0.061			1.807 (0.948–3.445)	0.072		
AFP (ng/mL)	0.822 (0.478–1.412)	0.478			0.861 (0.501–1.482)	0.590		
CEA (ng/mL)	1.507 (0.817–2.777)	0.189			1.178 (0.640–2.168)	0.598		
CA199 (U/L)	1.476 (0.872–2.497)	0.147			1.677 (0.992–2.835)	0.054		
CA724 (U/L)	2.068 (1.215–3.518)	0.007	1.722 (0.969–3.059)	0.064	1.880 (1.102–3.206)	0.020	1.675 (0.930–3.017)	0.085
CA125 (U/L)	2.267 (1.262–4.075)	0.006	1.743 (0.882–3.444)	0.110	2.838 (1.549–5.201)	0.001	1.920 (0.977–3.776)	0.059
BMI (kg/m^2^)	0.577 (0.342–0.973)	0.039	0.842 (0.442–1.604)	0.600	0.520 (0.307–0.883)	0.016	1.002 (0.521–1.929)	0.995
Age (< 53.50 vs. ≥53.50)	1.019 (0.589–1.763)	0.945			1.294 (0.752–2.228)	0.352		
Sex (male vs. female)	1.170 (0.633–2.216)	0.617			1.178 (0.638–2.176)	0.600		
TNM stage (I + II vs. III + IV)	0.628 (0.249–1.583)	0.324			0.640 (0.254–1.615)	0.345		
SMI (yes vs. no)	0.393 (0.231–0.669)	0.001			0.373 (0.219–0.636)	<0.001		
SFA (low group vs. high group)	0.454 (0.221–0.932)	0.031			0.589 (0.286–1.215)	0.152		
VFA (low group vs. high group)	1.101 (0.605–2.002)	0.753			1.134 (0.615–2.093)	0.687		
TFA (low group vs. high group)	0.814 (0.454–1.459)	0.489			0.987 (0.546–1.786)	0.966		
PNI (<48.13 vs. ≥48.13)	0.569 (0.328–0.989)	0.045			0.695 (0.400–1.205)	0.195		
PNI‐SAF (0 vs. 1 vs. 2)	Reference	0.012	Reference	0.272	Reference	0.046	Reference	0.530
	0.544 (0.284–1.006)	0.052	1.754 (0.673–4.573)	0.250	0.541 (0.293–1.000)	0.050	0.931 (0.350–2.475)	0.885
	0.351 (0.165–0.747)	0.007	0.721 (0.298–1.743)	0.468	0.452 (0.211–0.969)	0.041	0.604 (0.247–1.482)	0.271
PNI‐SMI (0 vs. 1 vs. 2)	Reference	0.018	Reference	0.055	Reference	0.076	Reference	0.067
	0.589 (0.307–1.132)	0.113	0.508 (0.255–1.011)	0.054	0.695 (0.364–1.327)	0.270	0.509 (0.252–1.030)	0.060
	0.314 (0.141–0.700)	0.005	0.274 (0.089–0.846)	0.024	0.392 (0.174–0.883)	0.024	0.280 (0.088–0.891)	0.031

### 3.4 OS and PFS for BMI, SMI, SFA, and PNI

**FIGURE 1:**
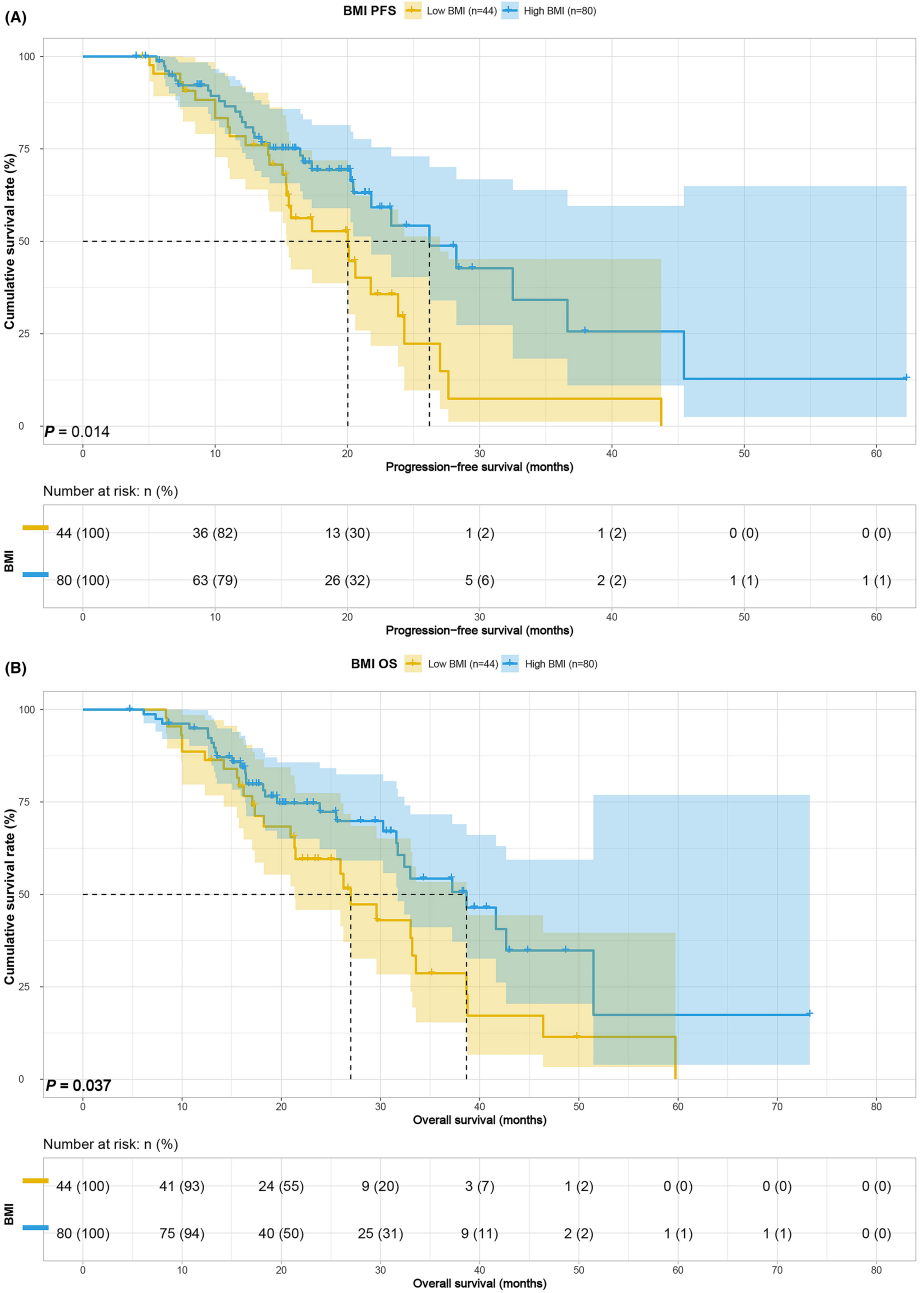
BMI related survival curve for (A) PFS and (B) OS.

**FIGURE 2:**
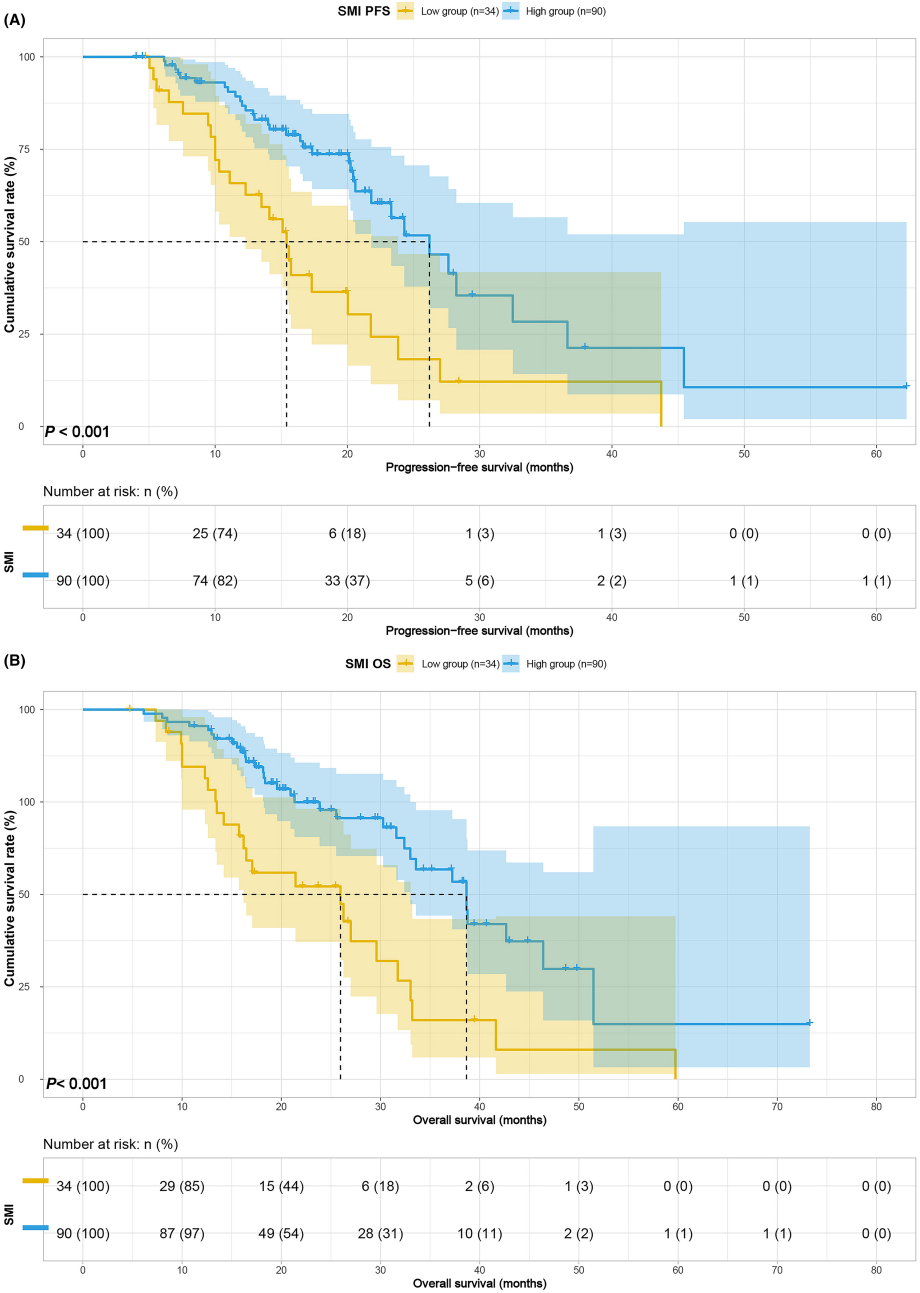
SMI related survival curve for (A) PFS and (B) OS.

**FIGURE 3:**
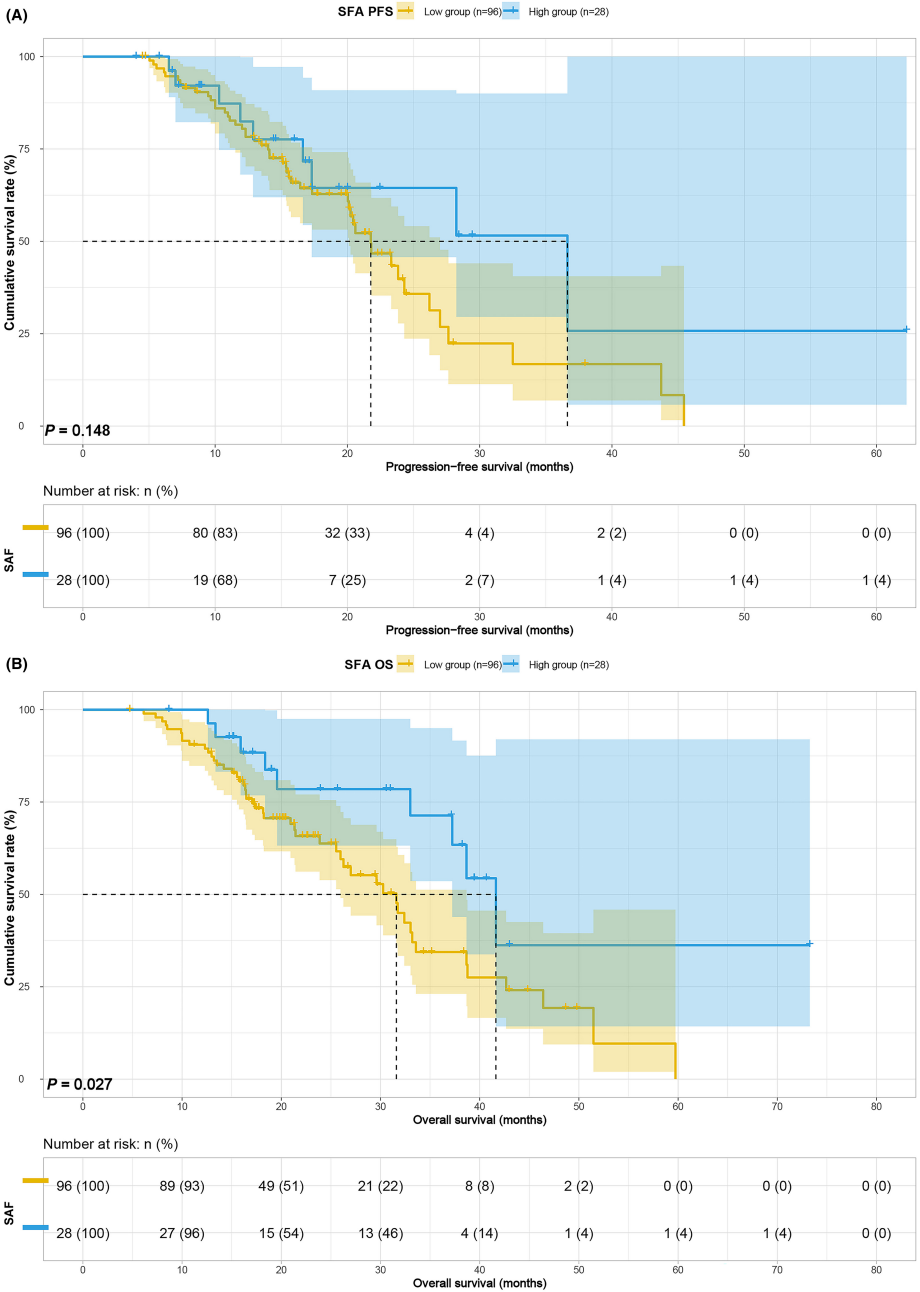
SFA related survival curve for (A) PFS and (B) OS.

**FIGURE 4:**
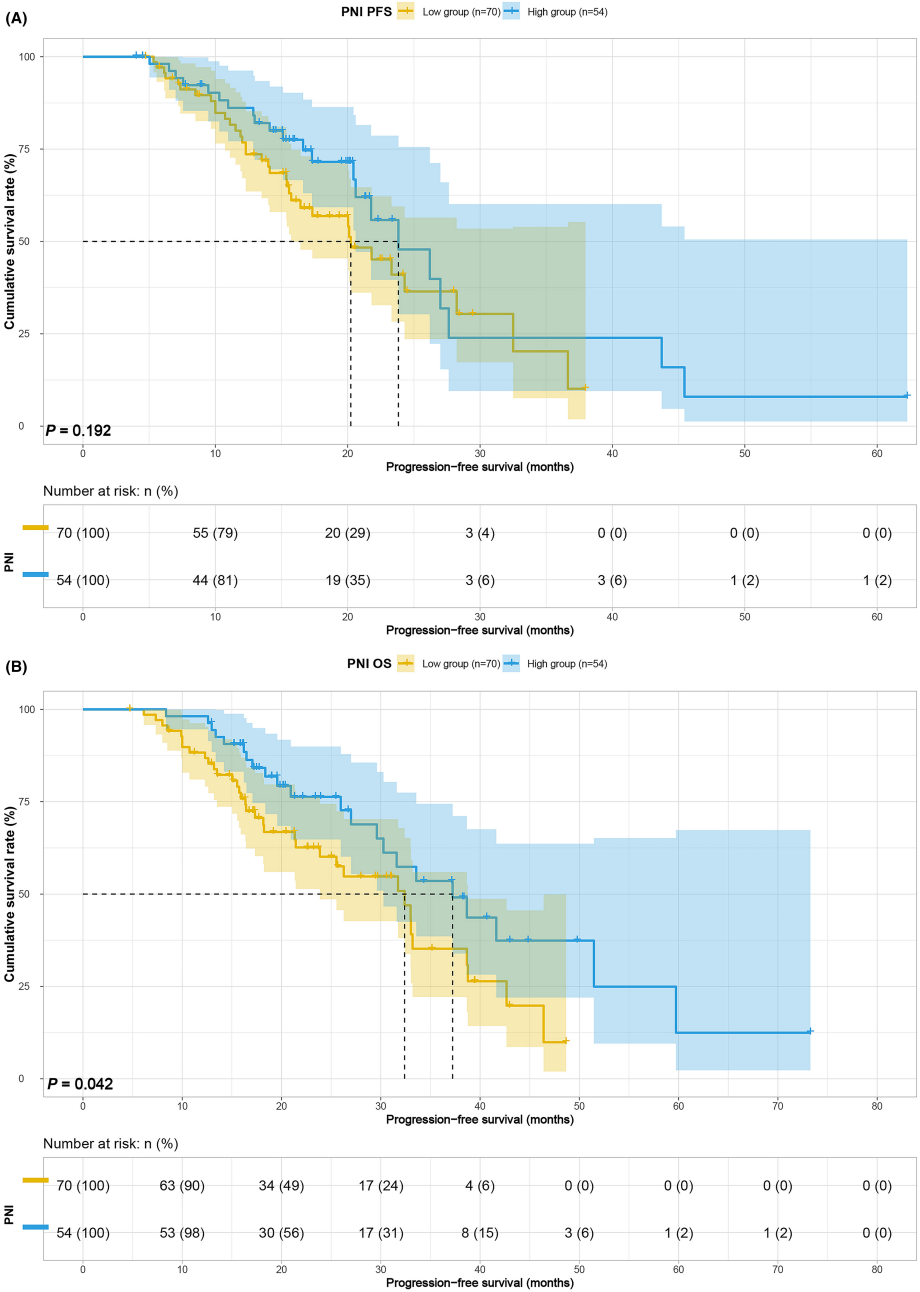
PNI related survival curve for (A) PFS and (B) OS.

Survival analysis based on body composition and blood indices revealed that higher BMI was associated with improved PFS (median, 20.03 vs. 26.20 months, *p* = 0.014) and OS (median, 27.00 vs. 38.67 months, *p* = 0.037) (Figure 1). Conversely, lower SMI was linked to poorer PFS (median, 15.40 vs. 26.20 months, *p* < 0.001) and OS (median, 25.97 vs. 38.67 months, *p* < 0.001) (Figure 2). Patients with lower SFA exhibited inferior PFS (21.77 vs. 36.63 months, *p* = 0.148) and OS (median, 31.60 vs. 41.63 months, *p* = 0.027) (Figure 3), and those with lower PNI demonstrated worse PFS (20.23 vs. 23.83, *p* = 0.192) and OS (32.40 vs. 37.23, *p* = 0.042) (Figure 4). Although SFA and PNI did not attain statistical significance in predicting PFS, their median survival exhibited a notable difference.

### 3.5 Survival analysis for PNI‐SFA and PNI‐SMI

**FIGURE 5:**
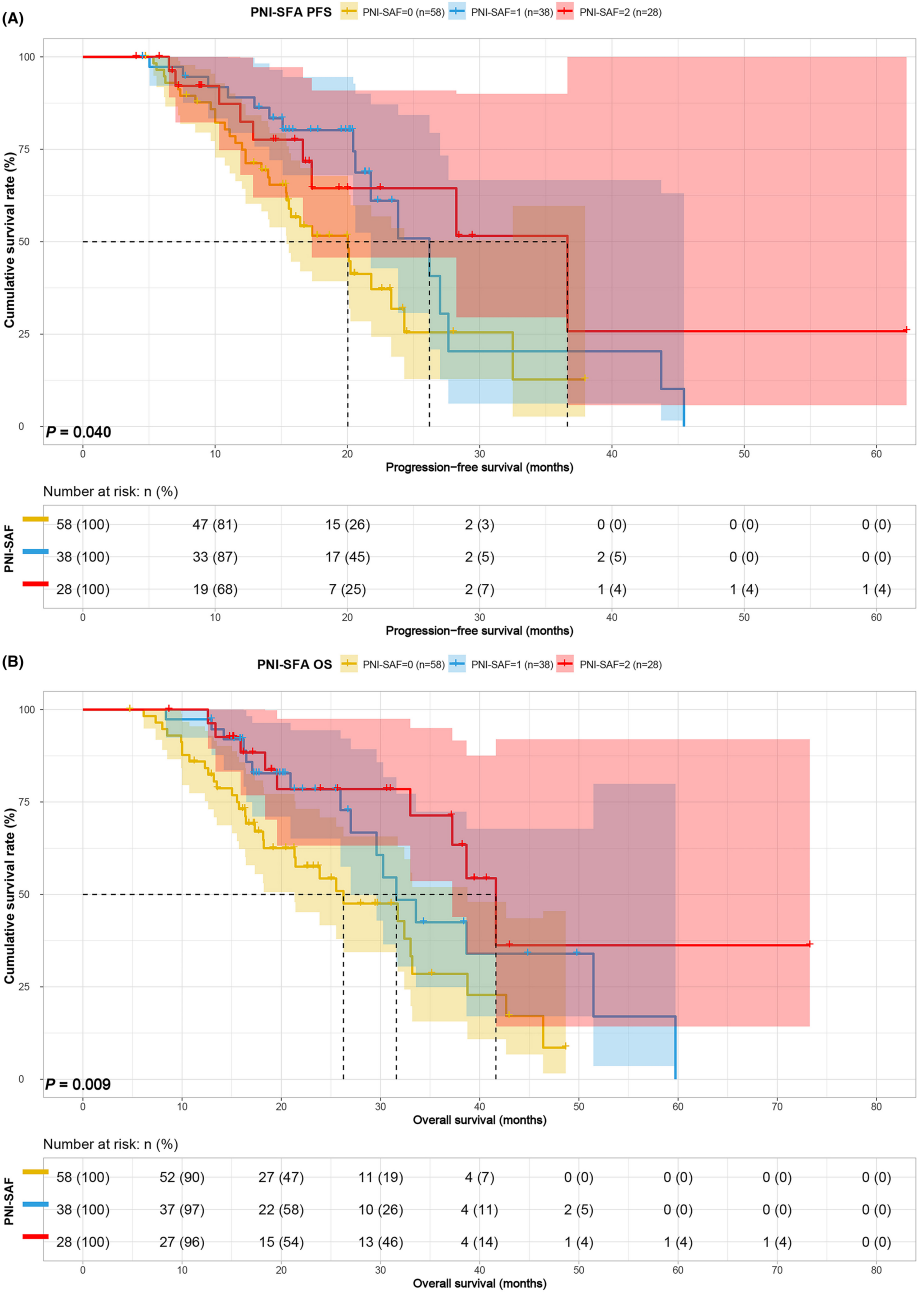
PNI‐SFA related survival curve for (A) PFS and (B) OS.

**FIGURE 6:**
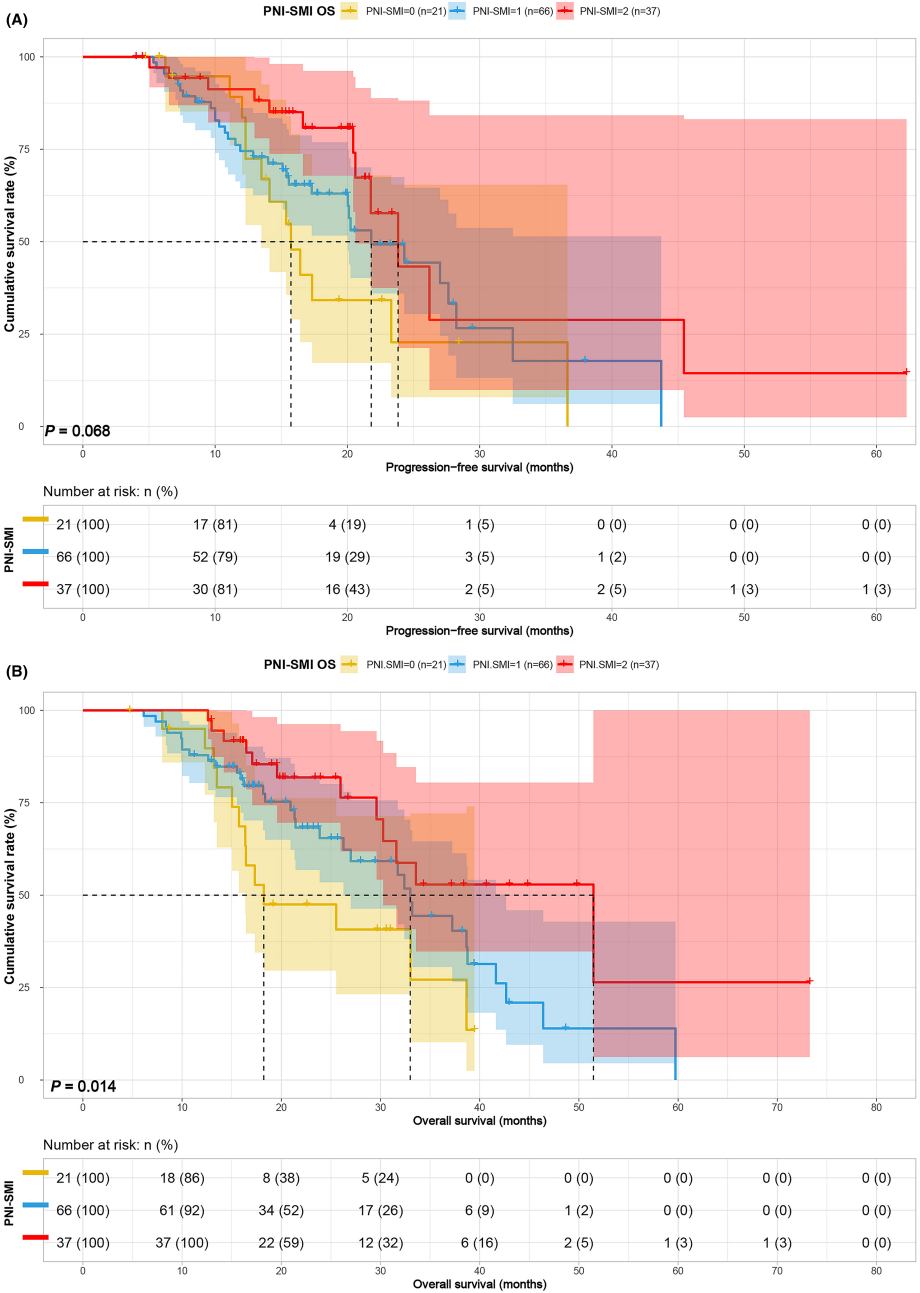
PNI‐SMI related survival curve for (A) PFS and (B) OS.

**FIGURE 7:**
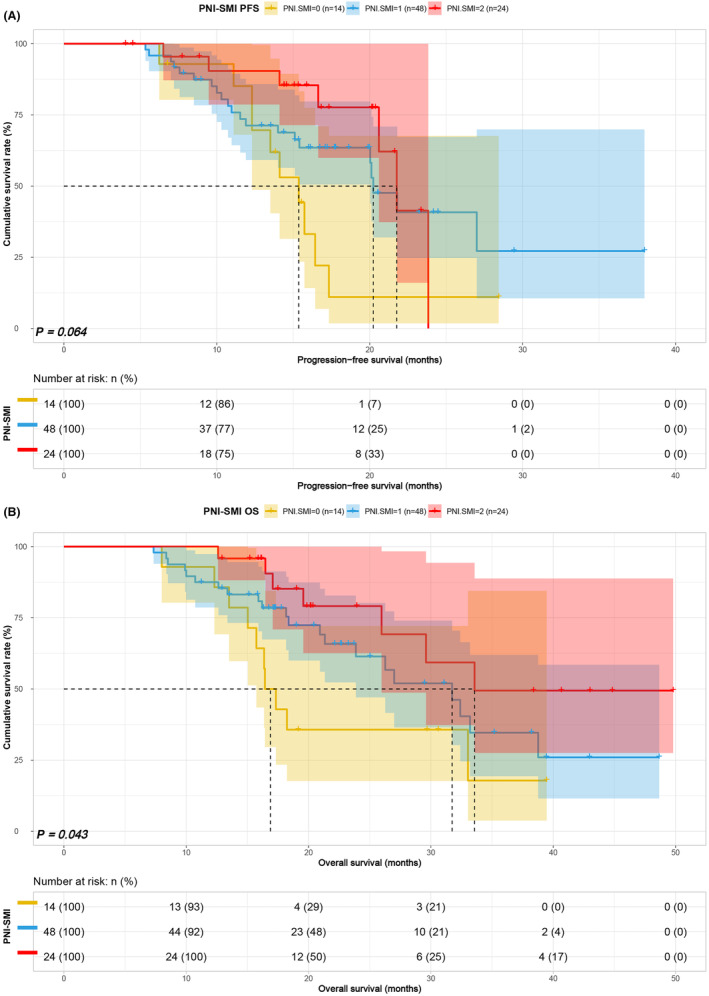
PNI‐SMI related survival curve for (A) PFS and (B) OS in IV stage patients.

Considering PNI as a common nutritional indicator, an in‐depth analysis of PNI combined with SFA and SMI was performed to differentiate the prognosis of patients in various score groups. In the PNI‐SFA group, patients in Group 1 and Group 2 displayed shorter PFS (20.03 vs. 26.20 vs. 36.63 months, *p* = 0.040) and OS (26.27 vs. 31.60 vs. 41.63 months, *p* = 0.009) compared to patients in Group 3 (Figure 5). Similarly, in the PNI combined with SMI group, patients in Group 1 and Group 2 exhibited poorer PFS (15.73 vs. 21.80 vs. 23.83 months, *p* = 0.068) and OS (18.23 vs. 33.00 vs. 51.47 months, *p* = 0.014) than patients in Group 3 (Figure 6). Notably, PNI‐SMI seemed to exhibit an inconsistent performance in predicting PFS compared to PNI‐SFA. The prognostic significance of PNI‐SMI in patients treated with ICIs was confirmed as an independent factor. To assess its sensitivity, we conducted a subgroup analysis focusing on stage IV patients. Our findings demonstrated that PNI‐SMI reliably predicted overall survival (OS) and progression‐free survival (PFS) among patients diagnosed with stage IV GC, aligning with previous observations (Figure 7).

### 3.6 Nomograms

**FIGURE 8:**
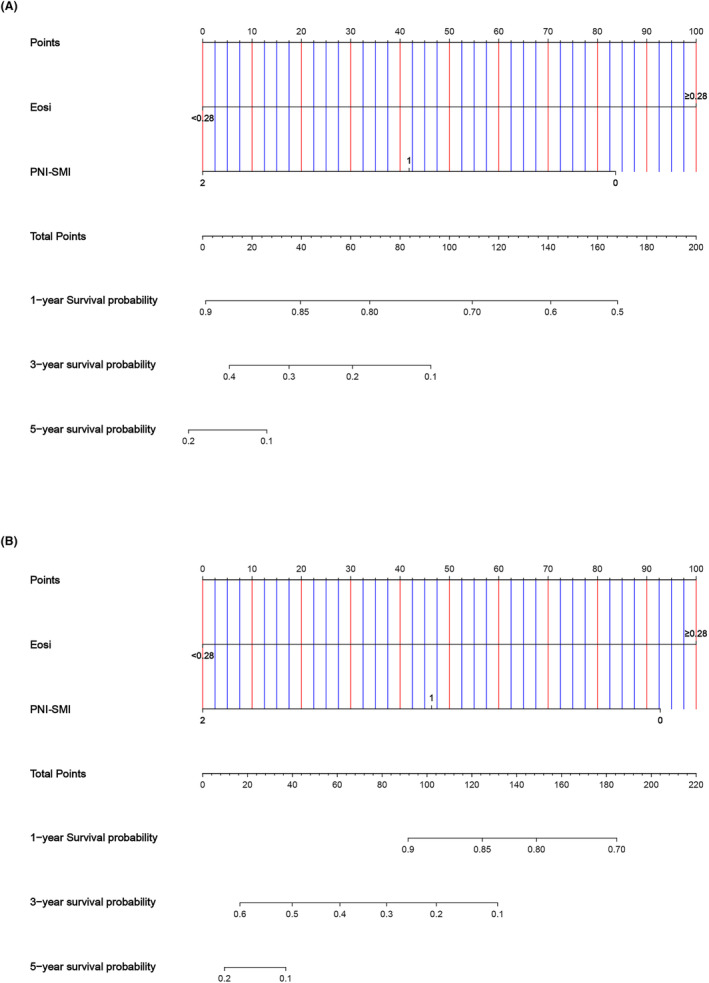
Nomogram for predicting 1‐, 3‐ and 5‐year survival probability of PFS (A) and OS (B).

Predictive models for PFS and OS were developed based on multivariate results. Independent prognosticators PNI‐SMI and Eosi were incorporated into nomograms for both PFS and OS (Figure 8).

## 4 DISCUSSION

In recent years, immunotherapy has emerged as a promising therapeutic avenue for various cancers, with monoclonal antibodies targeting immune checkpoints, such as PD‐1 and CTLA‐4, demonstrating clinical efficacy across diverse tumor types. This includes melanoma, non‐small‐cell lung cancer, renal cell carcinoma, bladder cancer, colorectal cancer, and others, fundamentally transforming the landscape of medical oncology.^21^ However, despite the favorable outcomes observed in some patients, a substantial proportion does not exhibit a sustained response to ICIs, leading to disease progression.^22^ This heterogeneity of response is often attributed to individualized immune resistance, which may be host‐related.^23^ Host‐related parameters, including BMI, play a significant role in influencing the outcomes of ICIs treatment.^24^ Contrary to expectations, clinical data analysis reveals that obese patients may derive greater benefits from ICIs compared to those with normal BMI, highlighting the limitations of BMI in accurately discerning abnormalities in abdominal fat distribution.^25^, ^26^, ^27^, ^28^, ^29^, ^30^

The intricate relationship between obesity and cancer has been well‐documented, with adipose tissue acting as a source of adipokines and inflammation‐related factors, contributing to disrupted energy homeostasis and disease progression.^31^, ^32^ Distinct abdominal fat distribution has been linked to the prognosis of various malignancies.^32^, ^33^ Studies have demonstrated the association between visceral adipose tissue accumulation and the development and progression of early‐stage colon cancer.^34^ Muscle mass, another indicator of nutritional status, has also been correlated with survival outcomes in cancer patients.^35^ Recognizing the importance of nutrition in cancer prevention, treatment, and survival,^36^ the PNI has been extensively studied and shown to predict the prognosis of GC patients.^37^, ^38^, ^39^, ^40^ However, the limitations of individual indicators, such as the influence of multiple factors on albumin and lymphocytes in PNI, necessitate a comprehensive assessment incorporating potential body composition prognostic indicators (SFA, SMI) to accurately determine the prognosis of GC patients undergoing ICIs treatment.^41^, ^42^

SMI has been extensively investigated within the GC research domain. For instance, studies such as that conducted by Feng‐Min Zhang et al. have developed and validated predictive models, specifically column charts, aimed at assessing muscle mass and radiodensity in GC patients at various stages.^43^ Additionally, Qian‐Tong Dong et al. conducted a comprehensive analysis exploring the relationship between body composition parameters, muscle strength, physical functioning, and their impact on postoperative complications and survival rates following radical gastrectomy for GC.^44^ Furthermore, Guang‐Tan Lin et al. investigated the body parameter SMI as an independent risk factor for tumor regression in patients treated with neoadjuvant chemotherapy combined with immunotherapy.^45^

Our study utilized Kaplan–Meier analysis, revealing that high PNI‐SFA and PNI‐SMI groups correlated with prolonged PFS and OS compared to their low PNI‐SFA and PNI‐SMI counterparts. Notably, PNI‐SMI exhibited a comparatively diminished predictive capacity for PFS compared to PNI‐SFA. Positive correlations with PFS and OS were observed for BMI, SFA, and SMI, while VFA and TFA did not emerge as prognostic indicators. Multivariate analysis identified PNI‐SMI and Eosi as independent prognostic factors for both PFS and OS. The establishment of an immune prediction model facilitated prognosis prediction based on PFS and OS column‐line graphs.

Despite the insights gained, the study has limitations, primarily its retrospective, single‐center nature, necessitating validation in a multicenter setting. Additionally, the inclusion of patients receiving different immunotherapy regimens introduces potential efficacy variations, requiring validation in cohorts with uniform treatment regimens. This study has encountered several limitations in its statistical methodology. First, the relatively small sample size led to a limited ROC curve in determining the cutoff value of SMI. Furthermore, the Kaplan–Meier method may yield unstable estimates with small sample sizes and a low number of events. Additionally, the Cox proportional hazards model permits only the comparison of relative risks and does not directly estimate absolute risks. This study contributes novel perspectives on hematological and body composition binding indices and the prognostic impact of fat area in GC patients receiving ICIs, offering innovative approaches for refining immune prediction models through comprehensive analyses of body composition changes, nutritional status, and inflammatory markers.

## 5 CONCLUSION

In summary, the amalgamation of the PNI with body composition indicators, specifically SMI and SAF, effectively reflects the nutritional status of individuals. Lower scores in PNI‐SMI and PNI‐SAF are associated with poorer clinical outcomes for patients. This underscores the potential utility of integrating body composition alterations and nutritional indicators as a meaningful biomarker for evaluating the prognosis of GC patients undergoing treatment with Immune ICIs.

## AUTHOR CONTRIBUTIONS

**Guiming Deng:** Writing – original draft (equal); writing – review and editing (equal). **Dayong Zhu:** Data curation (equal); investigation (equal); methodology (equal); supervision (equal). **Zhongze Du:** Data curation (equal); investigation (equal). **Yingwei Xue:** Project administration (equal); resources (equal). **Hongjiang Song:** Funding acquisition (equal); project administration (equal); resources (equal). **Yuanzhou Li:** Project administration (equal); resources (equal).

## FUNDING INFORMATION

Clinical Research Foundation of Wu Jieping Medical Foundation (No. 320.6750.2022‐07‐13).

## CONFLICT OF INTEREST STATEMENT

The authors declare no competing financial interests.

## ETHICS STATEMENT

This study was approved by the ethics committee of Harbin Medical University Cancer Hospital. All patients provided written informed consent before the study.

## Data Availability

The authors promise to provide the original data supporting this study without reservation.
